# Phosphoric Acid Etch Partially Restores the Initial Bond Strength of Composite to Silver Diamine Fluoride–Treated Enamel Using Universal Adhesives

**DOI:** 10.3390/dj11070161

**Published:** 2023-06-28

**Authors:** Zaher Jabbour, Mijoo Kim, Marc Hayashi, Reuben Kim

**Affiliations:** 1 Section of Interdisciplinary Dentistry, School of Dentistry, University California Los Angeles, Los Angeles, CA 90095, USA; 2 Restorative Materials Research Laboratory, Section of Restorative Dentistry, School of Dentistry, University California Los Angeles, Los Angeles, CA 90095, USA; mijookim@dentistry.ucla.edu (M.K.); mhayashi@dentistry.ucla.edu (M.H.); rkim@dentistry.ucla.edu (R.K.)

**Keywords:** silver diamine fluoride, phosphoric acid etch, composite bond, enamel

## Abstract

Background: Restoring bonding composite to silver diamine fluoride (SDF)-treated enamel is challenging. This study investigates if phosphoric acid etch restores composite bond strength to SDF-treated enamel using universal adhesives. Methods: Twenty-four recently extracted permanent teeth were randomly divided into 4 (2 experimental (SDF) and 2 control (CTR)) groups: SDF+Water: SDF (1 min) then water rinse (15 mL); CTR+Water: no treatment and water rinse (15 mL); SDF+Etch+Water: SDF (1 min), 35% phosphoric acid (40 s) then water rinse (15 mL); CTR+Etch+Water no treatment, 35% phosphoric acid (40 s) then water rinse (15 mL). The enamel surface in all the groups was bonded (All-Bond Universal) to 4–5 mm composite blocks (Z-250). Each sample was sectioned, and 6–8 beams (1 mm × 1 mm) were selected. The micro-tensile bond strength was measured by dividing the micro-tensile force peak by the adhesive surface area. Univariate ANOVA and Chi-square were used for between-group comparisons with *p* < 0.05. Results: SDF+Water had significantly lower tensile strength compared to all the groups (*p* < 0.05). Although no difference was found in the tensile strength between the SDF+Etch+Water and the CTR+Etch+Water, the SDF+Etch+Water had significantly more adhesive failures compared to the CTR+Etch+Water (*p* = 0.047). Conclusions: While phosphoric acid etch seems to restore the initial composite bond strength to SDF-treated enamel, the long-term success of composite restorations bonded to SDF-treated enamel may need further investigation.

## 1. Introduction

Dental caries is considered one of the detrimental factors which can compromise oral health throughout the lifetime of both children and adults. The presence of untreated dental caries on permanent teeth was the most prevalent health condition in the world in 2019 [1]. In the same year, the prevalence of caries in primary teeth was the highest of all diseases in children aged 0 to 14 years old [1]. 

The treatment of dental caries historically has focused on the surgical removal of the infected tooth structure and its replacement with restorative materials. As a result, different protocols for amalgam, composite resin, and glass ionomer restorations were developed. In 2014, silver diamine fluoride (SDF) was approved by the United States Food and Drug Administration as a desensitizing agent. SDF has also shown to be able to arrest or slow the progression of carious lesions [2].

Despite the success of SDF as a conservative nonsurgical modality to slow or arrest the progression of carious lesions, a recent randomized controlled clinical trial demonstrated that composite restorations bonded to sound caries-free enamel of permanent molars treated with 38% SDF indirect pulp capping led to significantly higher marginal stain at 3-, 6-, and 12-month follow-ups compared to non-SDF-treated teeth [3]. Evidence reported in the literature suggests that bonded restorations with well-sealed margins are keys to prevent contamination and halt the progression of carious lesions [4,5]. Mertz-Fairhurst et al. [6] demonstrated that placing a bonded well-sealed restoration over frank cavitated carious dentin lesions could arrest the clinical progression of the carious lesions for over 10 years. In general, the enamel bond protects the dentin bond and prevents micro-leakage and recurrent caries around the restorations [7]. As a result, the ability to bond composite restorations to enamel after SDF application is critical for the long-term success of the nonsurgical management of carious lesions.

Only a few studies in the literature have attempted to investigate the composite bond to enamel after SDF application [8,9,10]. On the other hand, many reports have demonstrated that SDF significantly impairs the bond strength of a composite to dentin through various mechanisms [11,12]. One mechanism is the high pH of SDF, which can interfere with the acidity of composite bonding agents [8]. Two recent meta-analyses indicated that a rinsing step after SDF application could improve the bond strength of a composite resin to dentin [11,12]. However, these meta-analyses did not include analyses for the enamel bond or subgroup comparisons to account for different protocols for rinsing SDF. Only one study [13] included groups for comparing water rinse and acid etch and found significant differences between the groups when using 12% SDF but not 38% SDF. 

In addition, studies that evaluated the bond strength of a composite to SDF-treated enamel used the shear bond strength test. Although the shear bond strength test can be performed predictably on intact uniform flat surfaces, the test could result in a concentration of stress over a narrow area of the tooth substrate [14]. This is manifested by highly nonuniform stress distribution and fracture initiation in the specimen. As a result, cohesive failure in the substrate (i.e., tooth) is often observed when using the shear bond strength test leading to severe underestimation of the stress level that the specimen can resist before fracture [15]. Contrarily to the shear bonding strength test, the micro-tensile bond strength test leads to a better distribution of forces, especially when the surface area is small [14]. However, the test is technique-sensitive and requires sectioning of the samples into stick-shaped beams using a precision cutting machine. This might lead to damage and/or dehydration of the specimens [14]. Therefore, the aim of this study was to compare the impact of phosphoric acid etch and water rinse on the bond strength of composite resin to enamel after SDF application using the micro-tensile bond strength test. The study null hypothesis stated that there is no difference in the bond strength of composite resin bonded to SDF-treated enamel after phosphoric acid etch and water rinse using the micro-tensile bond strength test.

## 2. Materials and Methods

This in vitro study was approval by the Institutional Review Boards (IRB) of the University of California Los Angeles (UCLA) (IRB #19-002271). The teeth used in this study were obtained from the UCLA Oral Maxillofacial Surgery clinic, according to the protocol approved by UCLA IRB as Exempt #4. In our institution, this exemption does not require patient consent, since the specimens were not identified and will not be reidentified after the experiment. Only intact young adult molars and premolars were included in the study. The teeth were likely extracted due to malposition or orthodontic reasons. All the teeth were inspected under dental loupes (3×) magnification. Teeth with any cracks or loss of structure due to various reasons such as the presence of caries, restorations, abrasion, attrition, erosion, or abfraction were excluded from the study.

### 2.1. Sample Preparation

A total of 24 intact permanent teeth (8 premolars and 16 molars) were used for the micro-tensile bond strength test (μTBS). The teeth were extracted 3–4 months prior to the study. Briefly, immediately after extraction, the teeth were stored in a 0.1% thymol solution. The teeth were then cleaned, and any debris or calculus was removed. After that, the teeth were placed in a fresh solution of 0.1% thymol and stored at 4 °C. About 48–72 h prior to conducting the testing procedures and throughout the experiment, the teeth were stored in Hank’s balanced salts solution (HBSS) and kept at 4 °C [16,17]. 

The teeth were randomly divided into 4 groups: 2 experimental groups (SDF) and 2 control groups (CTR). Each group included 6 teeth (2 premolars and 4 molars), as a minimum sample size of 5 teeth per group was previously suggested [16]. The groups were as follows: Group 1 (SDF+Water), Group 2 (CTR+Water), Group 3 (SDF+Etch+Water), and Group 4 (CTR+Etch+Water).

**Figure 1:**
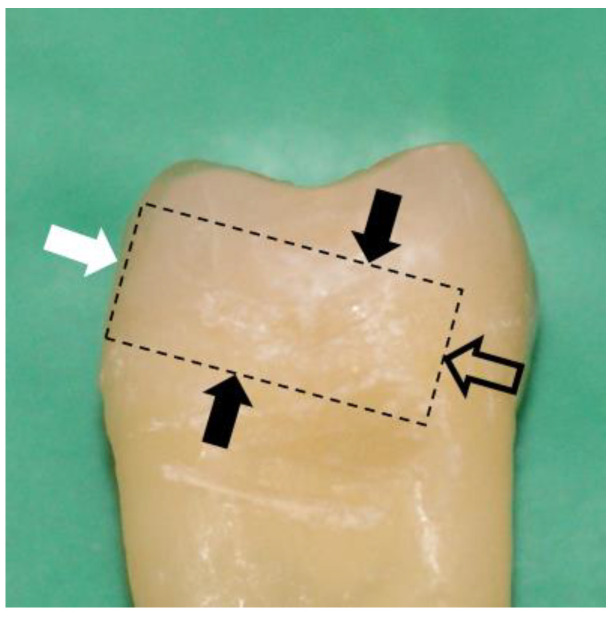
The superficial enamel (white arrow) was flattened and polished with silicone carbide abrasive sandpaper P320 under running water. The samples were sectioned perpendicular to the flattened buccal or lingual surfaces (solid black arrows). Using the mounting surface (outlined black arrow), the teeth were attached to a cylindrical acrylic support so that the composite buildups and μTBS were performed perpendicular to the flattened buccal or lingual enamel surface (white arrow).

The superficial buccal or lingual enamel of the teeth was flattened and polished with silicone carbide abrasive sandpaper P320 grit using circular motion for 1 min under running water [16,17]. Minimal superficial enamel (<0.05 mm) was removed to obtain a flat surface (around 5 × 5 mm) and avoid exposure of the dentin [17]. Since the thickness of enamel is not uniform across on the buccal or lingual surface of teeth (enamel is thickest close to the cusp tips and thinnest close to the cement–enamel junction), more coronal enamel was removed compared to cervical enamel. All the samples were sectioned perpendicular to the buccal or lingual flattened enamel surface (Figure 1) [17] and mounted onto a cylindrical acrylic support so that the composite buildups and μTBS tests could be performed perpendicular to the flattened buccal or lingual enamel surface (Figure 1). The enamel surface of each group received a different protocol as below. The materials used in this study are listed in Table 1 [18,19,20].

Group 1 (SDF+Water): SDF was applied on all the samples in this group for 1 min, dried with a gentle air stream for 1 min (distance about 1 cm) following the manufacturer recommendations [21], and rinsed with 15 mL water. 

Group 2 (CTR+Water): The samples in this group received no SDF treatment but were only rinsed with 15 mL water. 

Group 3 (SDF+Etch+Water): The samples in this group received the same protocol as Group 1 except that the enamel was etched with 35% phosphoric acid after SDF. Phosphoric acid was applied for 40 s, agitated using micro-brush [22,23], rinsed with 15 mL water, and dried with a gentile air stream for 10 s. 

Group 4 (CTR+Etch+Water): The samples in this group received the same protocol as the samples in Group 2 except that the 35% phosphoric acid etch was applied for 40 s and then rinsed with 15 mL water.

The enamel surface of all the samples was then coated with 2 layers of All-Bond Universal (BISCO, Schaumburg, IL, USA) by scrubbing the flattened enamel surface with a microbrush for 10–15 s per coat, then dried for 10 s, and light cured for 10 s with an intensity of 1200 (±10%) mW/cm^2^ (Paradigm™ DeepCure Curing Light, 3M, Saint Paul, MN, USA) [24] according to the manufacturer’s recommendations for direct restorations [25]. After curing, 2 mm composite increments (Z-250, shade A1, 3M, Saint Paul, MN, USA) were applied and light-cured for 40 s to form 4–5 mm blocks with the help of a clear matrix band. All the specimens were kept under 100% humidity and room temperature for 24 h.

### 2.2. Micro-Tensile Bond Strength (μTBS) Test

The specimens were sectioned into 1 mm × 1 mm beams using a low-speed precision cutting machine equipped with diamond blade (Isomet, Buehler, Lake Bluff, IL, USA) [16]. A total of 6–8 beams were selected from each sample, and a μTBS test was performed 48–72 h after preparation as previously described [26]. Briefly, beams were fixed onto each of the jaws of the micro-tensile tester machine (BISCO Inc., Schaumburg, IL, USA) using cyanoacrylate cement (Zapit; DVA, Corona, CA, USA) and separated at a speed of 1 mm/min [26]. The device was previously calibrated by the manufacturer. Each jaw was marked for the standardized reproducible placement of beams. The interface between the jaws possesses a 2-mm-long notched space which permits placement of the beams at the enamel–composite interface [26]. In addition, the two jaws are equipped with a track that allows each jaw to predictably be repositioned and ensures movement of each jaw in the opposite direction on the micro-tensile machine tester with negligible friction [26]. The surface area at the enamel–composite interface was measured using a digital caliper. The peak micro-tensile force was registered, and the tensile strength was recorded by dividing the recorded peak number by the adhesive surface area for each beam (MPa) [26]. The specimens were initially examined by dental loupes (3×) magnification to determine the validity of the test (e.g., glue failure). High resolution digital macrographs, which allow >20× magnifications, were then taken to document the failure mode of each beam, which was categorized as adhesive failure, cohesive tooth failure, cohesive composite failure, or mixed failure [27]. Adhesive failures were failures that occurred at the interface between the enamel and the composite, while both the substrate tooth and the composite remained intact. Cohesive tooth failures and cohesive composite failures were failures that were entirely either in the tooth or in the composite buildups, respectively. Mixed failures were failures that involved both the tooth structure and composite buildups [16,27].

### 2.3. Statistical Analyses

The number of teeth samples and beams included in each group is shown in Table 2. A minimum sample size of 5 teeth per group was previously suggested [16]. Beams with pretest failure were treated as left-censored data and were replaced by the mean value between 0 MPa and the lowest value measured in the same experimental sample [16]. Beams with glue failure or manipulation errors were excluded. Since beams do not meet the independence assumption, and to account for within-sample correlation [28], the difference in the tensile bond strength was compared between the groups using two-way (group and tooth type) ANOVA with random effect for the sample [16,28]. Post hoc analyses were performed for between-group comparisons using Bonferroni adjustment. Between-group comparisons of frequency of failure mode were conducted using the Chi-square test. SPSS 28 and a significance level of *p* < 0.05 were used for all the analyses.

## 3. Results

The total number of beams was 168. Pretest failure was observed in four beams in the SDF+Water group and one beam in the CTR+Water group (Table 2). These beams were treated as left-censored data. Glue failure (any failure pathway involving glue [16]) was observed in one beam in the SDF+Water group and two beams in the CTR+Etch+Water group (Table 2). These beams were excluded from the study [16]. In addition, one beam was excluded due to manipulation error. As a result, the total number of beams included in the study was 164.

**Figure 2:**
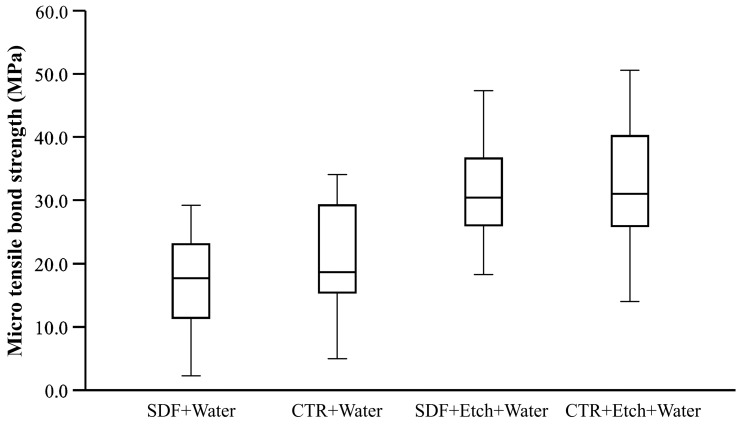
Box plot of raw data showing the tensile bond strength (MPa) of each of the study groups.

The bond strength of the beams was found to follow the normal distribution. The raw data of bond strength are presented in Figure 2. The mean surface area of beams at the adhesive interface was 0.995 mm^2^ ± SD 0.054 with no statistically significant difference between the study groups.

**Figure 3:**
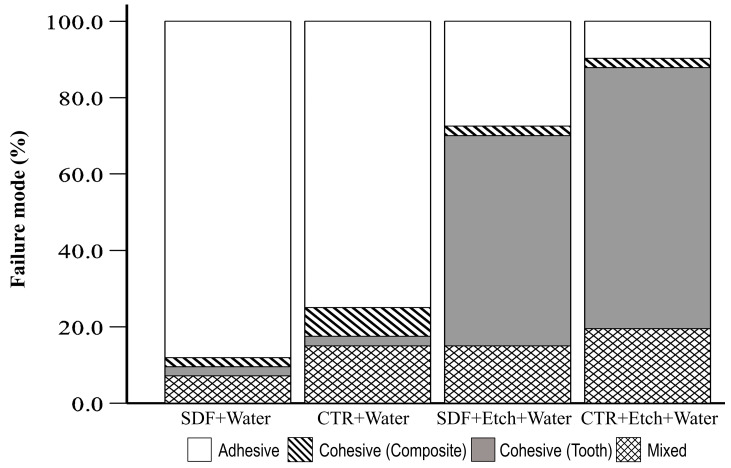
Mode of failure in each of the study groups.

**Figure 4:**
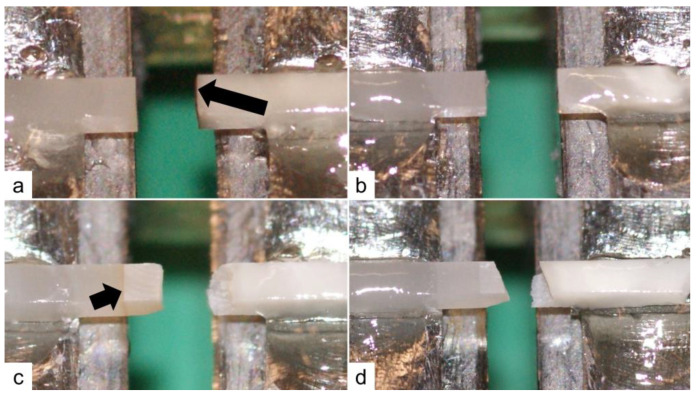
Macrographs showing the most frequent mode of failure in each of the study groups: (**a**) SDF+Water adhesive failure. Long black arrow showing remnants of SDF stain on enamel at the adhesive interface. (**b**) CTR+Water adhesive failure. (**c**) SDF+Etch+Water cohesive tooth failure. Short black arrow showing SDF stain at the enamel–composite interface with intact adhesion. (**d**) CTR+Etch+Water cohesive tooth failure.

Overall, the bond strength of the SDF+Water group was significantly lower than all the other study groups (Table 3). Adhesive failure was the most frequent method of failure in the SDF+Water and CTR+Water groups, with no difference in the frequency of failure between these two groups (Figure 3 and Figure 4, Table 4). However, the bond strength of beams with adhesive failure in the SDF+Water group was significantly lower when compared to beams with adhesive failure in the CTR+Water group (*p* = 0.012).

Samples treated with 35% phosphoric acid etch had significantly higher bond strength than the remaining study groups (Table 3), with no difference between the SDF+Etch+Water group and the CTR+Etch+Water group. However, although cohesive tooth failure was the most frequent mode of failure in the SDF+Etch+Water group (56.1%), adhesive failure (26.8%) occurred at a statistically higher frequency than adhesive failure in the CTR+Etch+Water group (9.8%) (Chi-square *p* = 0.047) (Figure 3). 

Since cohesive tooth failure with intact adhesive interface (Figure 3 and Figure 4, Table 4) was the most frequent method of failure in both the SDF+Etch+Water and the CTR+Etch+Water groups, the values of the actual bond strength to the enamel were likely higher than recorded.

## 4. Discussion

The use of SDF for the management of carious lesions is increasing [29]. As a result, clinicians will likely face the challenges of bonding composite resin to SDF-treated enamel and SDF-treated dentin. Although the impact of SDF on the bonding of a composite to dentin is well documented [11,12], this is the first study to evaluate the bond strength of a composite to enamel after SDF application using μTBS. A previous study reported that SDF impacted the bond stability of a composite to enamel as determined by the shear bond strength test and the fatigue bond strength test [8]. Another study reported no impact of SDF on bonding to enamel when the shear bond strength test was performed [9]. The difference between the two studies could presumably be explained by the etching step. This study supports previous research and meta-analyses showing that a rinsing step could improve the bond strength of a composite and that a 35% phosphoric acid etch is more effective than water rinse in restoring the composite bond strength. Therefore, the null hypothesis was rejected.

In our pilot experiments, a composite bonded to SDF-treated enamel without any rinse resulted in a very weak bond and a significantly high number of pretest failures (data not shown). One explanation could be the inhibition of the functional monomer 10-Methacryloyloxydecyl dihydrogen phosphate (10-MDP). 10-MDP interacts with hydroxyapatite using a phosphate group at one end and co-polymerizes with resin monomers through the methacrylate group at the other end [30]. This interaction with hydroxyapatite releases calcium and leads to the formation of 10-MDP-calcium salts and the self-assembly of 10-MDP monomers into nanolayers [31]. SDF Application could have blocked this process by increasing the acid resistance of the enamel due to its high pH (around 10–11) as well as the presence of silver and fluoride in the SDF and the formation of the silver phosphate and calcium fluoride layer [32]. Without a rinsing step and using only a self-etch technique, the presence of this layer likely prevented the exposure of hydroxyapatite and hindered the phosphate group in 10-MDP from chelating calcium ions in the hydroxyapatite; subsequently, it interfered with the 10-MDP bond and the formation of 10-MDP-calcium salt [33]. The current experiment suggests that, although water rinse seems to improve bond strength, it was not as strong when compared to samples without SDF application. Remnants of SDF were observed on the enamel surface of the failed adhesive interface in the SDF+Water group (Figure 4), which is consistent with previous in vitro reports [8,27]. Furthermore, stains at the enamel–composite interface were also observed in the SDF+Etch+Water group with intact adhesion interface (Figure 4). This is consistent with a recent randomized controlled clinical trial [3] demonstrating significantly higher marginal stains of composite restorations in groups treated with SDF at 3-, 6-, and 12-month intervals. However, our results suggest that marginal stains could appear as soon as few days following SDF application. 

On the other hand, dynamic application of 35% phosphoric acid etch after SDF application to enamel restored the bond strength of the composite to a level comparable to the control samples with etched enamel. Although no statistically significant difference was found in tensile bond strength, samples in the SDF+Etch+Water had significantly more frequent adhesive failure than CTR+Etch+Water (Figure 3). This suggests that although the bond strength of the SDF+Etch+Water group is comparable to that of the CTR+Etch+Water group, the actual bonding at the interface may have been compromised. Nonetheless, the high number of cohesive tooth fractures in the SDF+Etch+Water might imply that the initial bond strength could be clinically adequate.

In the present study, we used a self-etch bonding agent because it can be employed with or without 35% phosphoric acid etch. According to the manufacturer’s instructions, All-Bond Universal can be used in the self-etching technique and the total etching technique [25]. Although 15–30 s etch with 35% phosphoric acid was recommended by the manufacturer for a total etching technique of enamel without dentin [25], 35% phosphoric acid etch was applied in the present study for 40 s with a dynamic agitation motion [22]. It was previously reported that the bond strength of enamel with dynamic etch placement is stronger than a static application of the etch [23]. Dynamic application of the etch brings new acid, helps dislodge loosened islands of etched enamel, and opens the enamel prisms for bonding, which improves the overall bond strength of the composite [23]. Moreover, SDF is a highly basic solution, and application of acid etch helps neutralize the surface and could result in enamel etch. Furthermore, application of 35% phosphoric acid etch led to the formation of a yellow precipitate, likely silver phosphate [10], which is a water-insoluble salt that could be washed away with water rinsing. These three mechanisms could explain the ability of 35% phosphoric acid etch to restore the bond strength to enamel to an adequate level. Although All-Bond Universal is a self-etch adhesive with mild pH = 3.2, the amount of acid might not be enough to achieve adequate enamel etch in the presence of SDF. This likely led to high adhesive failure and low bond strength in the SDF+Water group. As shown in Figure 4, remnants of SDF were observed at the enamel–composite interface, which could act as a surface contaminant causing increased surface resistance to acid as well as 10-MDP inhibition and adhesive failure. 

The significantly higher frequency of adhesive failure in the SDF+Etch+Water group compared to the CTR+Etch+Water group is consistent with previous reports in dentin [27,34] indicating that the bond strength to dentin was not completely restored even after phosphoric acid etch, which resulted in more frequent adhesive failure than cohesive failure. These reports suggested that abrasive methods such as dental burs should be used in order to restore the bond strength to SDF-treated dentin. 

In this study, the enamel surface was flattened with the help of a low-speed precision cutting machine and silicone carbide abrasive sandpaper P320 [16]. It was previously reported that bonding to cut enamel is more predictable than uncut enamel [23]. In the present study, the enamel was flattened to ensure adequate μTBS. As a result, it is anticipated that a smear layer may have formed on the surface of the enamel. It was previously reported that the application of phosphoric acid etch could remove the smear layer [34]. In the present study, phosphoric acid etch was used with a self-etch bonding agent, as previous investigations suggested that application of self-etching agents alone failed to remove the smear layer, which led to significant reduction in bond strength [23]. This is consistent with our results showing significantly lower bond strength in the CTR+Water group when compared to the CTR+Etch+Water group. In addition, in the present experiment, composite buildups were bonded to the buccal or lingual enamel surface of the teeth samples, leading to the application of tensile forces perpendicular to the enamel prisms. It was previously reported that this configuration is associated with the highest μTBS compared to the composite being bonded to horizontal or longitudinal sections and forces oriented parallel to the enamel prisms [35]. Furthermore, although the outcomes of the μTBS and shear bond strength tests cannot be compared directly, our results for etched and non-etched enamel with 35% phosphoric acid seem reasonably within the range that the manufacturer reported using the shear bond strength test (total etch 38.0 ± 7.8; self-etched 26.2 ± 4.5) [36]. The dissimilarity in the bond strength could be attributed to the difference in surface area (current study 1 mm^2^; manufacturer 4.5 mm^2^) and to the concentration of forces over a narrow area leading to fracture initiation and underestimation of stress resistance when using the shear bond strength test [14,15].

Rinsing of SDF after application is a controversial topic. Previous publications suggested rinsing of SDF [37], whereas the manufacturer recommends not to rinse SDF [21]. A recent micro-CT study suggested that a rinsing step reduces the content of SDF in carious lesions [38]. However, the efficacy of SDF with or without a rinsing step is still to be determined in order to identify a protocol that maximizes the advantage of SDF and composite restorations.

Although this study suggests that the initial bond strength of a composite to SDF-treated enamel could lead to adequate clinical outcomes, the increased frequency of adhesive failure in the SDF-treated groups indicates that SDF could impact the long-term success of a composite bonded to enamel [8]. Restorations in the oral cavity are subjected to constant functional loading and thermal changes. As a result, future studies should attempt to study the ageing of a composite to enamel using thermocycling as well as the microleakage of restorations bonded to SDF-treated enamel. In addition, scanning electron microscope, elemental analyses, and micro-computed tomography should be used in order to visualize SDF at the interface between enamel and composite restorations.

## 5. Conclusions

Although the bond strength of a composite to SDF-treated enamel seems to be initially adequate with dynamic application of 35% phosphoric acid etch, the long-term success of composite restorations bonded to SDF-treated enamel may need further investigation.

## Figures and Tables

**Table 1:** Acidity and ingredients of products used in the study groups.

Product	pH	Ingredients	Manufacturer
Advantage Arrest (SDF 38%)	10–11	Silver diamine fluoride (Ag(NH_3_)_2_F) (30–50%); FD&C blue #1 (<1%); deionized water (≤62.5%)	Elevate Oral Care, West Palm Beach, FL, USA
All-Bond Universal (unit dose)	3.2	Bisphenol A diglycidylmethacrylate (20–50%); ethanol (30–50%); MDP (5–25%); 2-hydroxyethyl methacrylate (5–25%)	BISCO, Schaumburg, IL, USA
Ultra-Etch (Phosphoric acid etch 35%)	<1	Phosphoric acid (≥25–<40%); polyethylene glycol (1–10%); dimethicone (≥0.1–<10%); other	Ultradent, San Fernando, CA, USA

**Table 2:** Total number of beams included in each of the study groups.

Group	Premolar	Molar	Total Beams	Pre-Test Failure	Glue Failure	Manipulation Error	Total Beams Included
SDF+Water	2	4	43	4	1		42
CTR+Water	2	4	41	1		1	40
SDF+Etch+Water	2	4	41				41
CTR+Etch+Water	2	4	43		2		41

**Table 3:** Estimated mean tensile bond strength of study groups.

Group	Estimated	Standard Error	95% Confidence Interval	
	Mean		Lower Bound	Upper Bound
SDF+Water ^a^	16.34	1.14	14.08	18.60
CTR+Water ^b^	20.61	1.17	18.30	22.92
SDF+Etch+Water ^c^	31.90	1.16	29.62	34.18
CTR+Etch+Water ^c^	32.21	1.16	29.91	34.50

^a, b, c^ Statistically significant difference between groups (ANOVA with Bonferroni adjustment, *p* < 0.05).

**Table 4:** Number and percentage (%) of beams categorized by mode of failure and tooth type in each of the study groups.

Group	Tooth Type	Adhesive	Cohesive (Composite)	Cohesive (Tooth)	Mixed	Total	*p*
SDF+Water	Premolar	11 (91.7)	0 (0.0)	0 (0.0)	1 (8.3)	12 (100)	0.836
	Molar	26 (86.7)	1 (3.3)	1 (3.3)	2 (6.7)	30 (100)	
CTR+Water	Premolar	11 (78.6)	0 (0.0)	0 (0.0)	3 (21.4)	14 (100)	0.426
	Molar	19 (73.1)	3 (11.5)	1 (3.8)	3 (11.5)	26 (100)	
SDF+Etch+Water	Premolar	3 (25.0)	0 (0.0)	5 (41.7)	4 (33.3)	12 (100)	0.319
	Molar	8 (27.6)	1 (3.4)	17 (58.6)	3 (10.3)	29 (100)	
CTR+Etch+Water	Premolar	1 (7.7)	1 (7.7)	9 (69.2)	2 (15.4)	13 (100)	0.493
	Molar	3 (10.7)	0 (0.0)	19 (67.9)	6 (21.4)	28 (100)	

## Data Availability

Data are contained within the article.
